# CANDIDACY 2.0-UNRAVELING HEALTH INEQUITIES IN CHRONIC CONDITION CARE ACCESS

**DOI:** 10.1093/geroni/igae098.3816

**Published:** 2024-12-31

**Authors:** Sharon Koehn, C Allyson Jones, Claire Barber, Lisa Jasper, Anh Pham, Cliff Lindeman, Neil Drummond

**Affiliations:** University of Alberta, Edmonton, Alberta, Canada; University of Alberta, Edmonton, Alberta, Canada; University of Calgary, Calgary, Alberta, Canada; University of Alberta, Edmonton, Alberta, Canada; University of Alberta, Edmonton, Alberta, Canada; College of Physicians and Surgeons of Alberta, Edmonton, Alberta, Canada; University of Alberta, Edmonton, Alberta, Canada

## Abstract

The Inverse Care Law, positing that those most in need of healthcare are least likely to receive it, continues to describe persistent health inequities among older adults with chronic conditions. This study introduces Candidacy 2.0 (Chronic Condition (CC)), an innovative framework designed to unravel the complex mechanisms of these inequities in healthcare access for diverse older adult populations. Using Critical Interpretive Synthesis, we analyzed qualitative and mixed methods literature on rheumatoid arthritis experiences across various populations, including racial and ethnic minorities, LGBTQI+ individuals, and those with disabilities. Our key finding was the identification of a crucial eighth dimension: the “embodied relational self,” which transforms the framework into a powerful tool for understanding intersectional experiences of health inequity. Candidacy 2.0 (CC) offers a comprehensive understanding of how patients’ and care providers’ experiences are shaped by systemic injustices and social determinants of health. By integrating approaches like intersectionality, concordance, and recursivity, this model provides a new lens to conceptualize and address health disparities in chronic condition management for older adults. The framework’s significance lies in its potential to guide the development of culturally relevant health promotion and intervention efforts, offering concrete explanations for access challenges rooted in socially patterned influences. Our findings have substantial implications for enhancing health equity in geriatric healthcare delivery, informing policy, and driving further research. By implementing Candidacy 2.0 (CC), stakeholders can develop targeted interventions that address the unique healthcare needs of diverse older adults, working towards eliminating disparities across the life course.

